# Balancing selection and recombination as evolutionary forces caused population genetic variations in golden pheasant MHC class I genes

**DOI:** 10.1186/s12862-016-0609-0

**Published:** 2016-02-18

**Authors:** Qian-Qian Zeng, Ke He, Dan-Dan Sun, Mei-Ying Ma, Yun-Fa Ge, Sheng-Guo Fang, Qiu-Hong Wan

**Affiliations:** The Key Laboratory of Conservation Biology for Endangered Wildlife of the Ministry of Education, State Conservation Center for Gene Resources of Endangered Wildlife, College of Life Sciences, Zhejiang University, Hangzhou, Zhejiang 310058 China; College of Animal Science and Technology, Zhejiang A&F University, Lin’an, Zhejiang 311300 China

**Keywords:** Balancing selection, *Chrysolophus pictus*, Galliformes, Genetic diversity, Major histocompatibility complex, MHC class I, Population genetics, Recombination

## Abstract

**Background:**

The major histocompatibility complex (MHC) genes are vital partners in the acquired immune processes of vertebrates. MHC diversity may be directly associated with population resistance to infectious pathogens. Here, we screened for polymorphisms in exons 2 and 3 of the *IA1* and *IA2* genes in 12 golden pheasant populations across the Chinese mainland to characterize their genetic variation levels, to understand the effects of historical positive selection and recombination in shaping class I diversity, and to investigate the genetic structure of wild golden pheasant populations.

**Results:**

Among 339 individual pheasants, we identified 14 *IA1* alleles in exon 2 (*IA1*-E2), 11 *IA1*-E3 alleles, 27 *IA2*-E2 alleles, and 28 *IA2*-E3 alleles. The non-synonymous substitution rate was significantly greater than the synonymous substitution rate at sequences in the *IA2* gene encoding putative peptide-binding sites but not in the *IA1* gene; we also found more positively selected sites in *IA2* than in *IA1*. Frequent recombination events resulted in at least 9 recombinant *IA2* alleles, in accordance with the intermingling pattern of the phylogenetic tree. Although some *IA* alleles are widely shared among studied populations, large variation occurs in the number of IA alleles across these populations. Allele frequency analysis across 2 *IA* loci showed low levels of genetic differentiation among populations on small geographic scales; however, significant genetic differentiation was observed between pheasants from the northern and southern regions of the Yangtze River. Both STRUCTURE analysis and F-statistic (*F*_*ST*_) value comparison classified those populations into 2 major groups: the northern region of the Yangtze River (NYR) and the southern region of the Yangtze River (SYR).

**Conclusions:**

More extensive polymorphisms in *IA2* than *IA1* indicate that *IA2* has undergone much stronger positive-selection pressure during evolution. Moreover, the recombination events detected between the genes and the intermingled phylogenetic pattern indicate that interlocus recombination accounts for much of the allelic variation in *IA2*. Analysis of the population differentiation implied that homogenous balancing selection plays an important part in maintaining an even distribution of MHC variations. The natural barrier of the Yangtze River and heterogeneous balancing selection might help shape the NYR-SYR genetic structure in golden pheasants.

**Electronic supplementary material:**

The online version of this article (doi:10.1186/s12862-016-0609-0) contains supplementary material, which is available to authorized users.

## Background

The major histocompatibility complex (MHC) is a primary factor in initiating immune defenses, and it is composed of glycoproteins that are specialized to present foreign antigens to T lymphocytes [1]. The MHC multigene family can be divided into at least 2 main classes of genes: class I and class II. Class I molecules are expressed in a wide variety of nucleated cells and mainly respond to intracellular parasites [2, 3]. MHC class I proteins are structurally composed of a cytoplasmic region, a transmembrane portion, and 3 extracellular domains (α1, α2, and α3), where antigen-binding domains (α1 and α2) encoded by exons 2–3 interact to form the hypervariable peptide-binding region (PBR) [2].

Apart from their prominent role in immune responses, much attention has been paid to MHC genes because of their extensive polymorphism. In recent years, the patterns of genetic variability in MHC class I genes have been intensively investigated across different vertebrate taxa, including mammals [4, 5], birds [6, 7], reptiles [8, 9], amphibians [10, 11], and fishes [12, 13]. The mechanisms that generate abundant MHC variation primarily involve parasite-related balancing selection [14], such as frequency-dependent selection or overdominant selection [15]. Because fast-evolving pathogens can easily escape the immune surveillance of common host MHC alleles, frequency-dependent selection prevails, contributing to the generation and retention of rare alleles [16]. Consequently, changes in the pathogen community over time and location lead to MHC variation in host populations [17]. In the case of overdominance, heterozygotes exhibiting superior recognition of a wider range of pathogens mount a stronger immune defense against pathogen infections than do homozygotes [18]. In addition, MHC-based mating preferences increase MHC gene heterogeneity in progeny [19–21].

In diverse vertebrate lineages, including birds, the MHC gene family has an extremely complex molecular architecture and genome organization with high gene copy numbers, abundant pseudogenes, frequent recombination, and homologous chromosome exchanges [22–26]. The complex evolutionary pattern in MHC genes appears to be dictated both by birth-and-death and concerted evolution [27]. In the birth-and-death mechanism, some novel duplicated genes remain functional for long periods in the genome, whereas others decay into non-functional genes (pseudogenes) or are completely eliminated from the genome [25, 28]. The concerted evolution model implies that different members of the MHC family evolve as a unit, primarily due to recombination across paralogous genes [27, 29]; as a result, genes within a species are more similar compared to orthologous genes in closely related species. Moreover, such widespread sequence homogeneity caused by repeated recombination events increases the difficulty in assigning alleles to specific MHC loci. Therefore, given the current poor understanding of MHC genomic organization, MHC single-locus typing in some non-model avian taxa is more challenging [7, 30–32]. The chicken MHC-B (*Gallus gallus*, Phasianidae, Galliformes), the first representative of the “minimal essential” MHC, is highly condensed and dramatically simpler than the mammalian MHC, containing only 2 classical class I genes, *BF1* and *BF2* [33, 34]. The detailed knowledge of MHC organization in chickens and its compressed nature facilitated the characterization of MHC diversity at the locus-specific level [35].

**Fig. 1:**
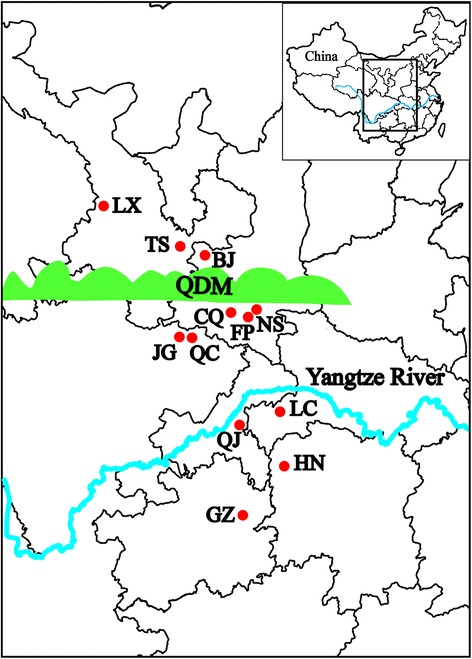
Geographic sampling locations of wild golden pheasants in China. The green zone represents the Qinling-Daba Mountain (QDM), which spans across central and western China, while the blue curve indicates the Yangtze River running from east to west China. Red dots mark all wild populations sampled for our study, and the population abbreviations are as follows: LX, Linxia; TS, Tianshui; BJ, Baoji; CQ, Changqing; FP, Foping; NS, Ningshan; JG, Jiange; QC, Qingchuan; LC, Lichuan; HN: Hunan; QJ: Qianjiang; GZ, Guizhou. This figure was created based on the map of China (https://commons.wikimedia.org/wiki/File%3AChina-map.xcf)

Golden pheasants (*Chrysolophus pictus*, Phasianidae, Galliformes), representing an endangered species in China, are scattered in the central and western regions of Mainland China [36]. Their main distribution areas are divided by 2 major geographical barriers: the Yangtze River and Qinling-Daba Mountain (QDM) (Fig. 1). To provide efficient protective strategies for this pheasant, we prioritized screening of MHC class I variation among wild populations. Previous studies on the golden pheasant MHC have reported the complete sequence of the gold pheasant MHC-B region, which is nearly identical to the chicken MHC-B gene arrangement [37]. The 97-kb golden pheasant MHC-B comprises 20 streamlined genes and conforms to the minimal essential hypothesis. In addition, only 2 duplicated functional class I genes (*IA1* and *IA2*) were identified in this species, as in chickens [37]. Based on these findings, we examined allelic variation of classical MHC class I genes in golden pheasants. The main objectives of this study were to (i) characterize sequence polymorphisms of exons 2 and 3 from both *IA1* and *IA2* genes through locus-specific PCR amplification, (ii) examine the role of historical positive selection and recombination in shaping class I diversity, and (iii) infer the population-genetics structure on the basis of MHC variation.

## Results

### MHC class I diversity

For the *IA1* and *IA2* genes, no more than 2 alleles were identified per pheasant by the PCR-based, single-stranded conformation polymorphism (SSCP) technique, indicating that single-locus genotyping was optimal using the present primer sets. For *IA1*, 23 of 254 (9 %) and 54 of 236 (23 %) nucleotide sites were variable at exons 2 and 3, and 14 and 11 alleles were identified, respectively. For *IA2*, 74 of 251 (29 %) and 71 of 253 (28 %) nucleotide sites were variable, and 27 and 28 alleles were found at exons 2 and 3, respectively.

**Fig. 2:**
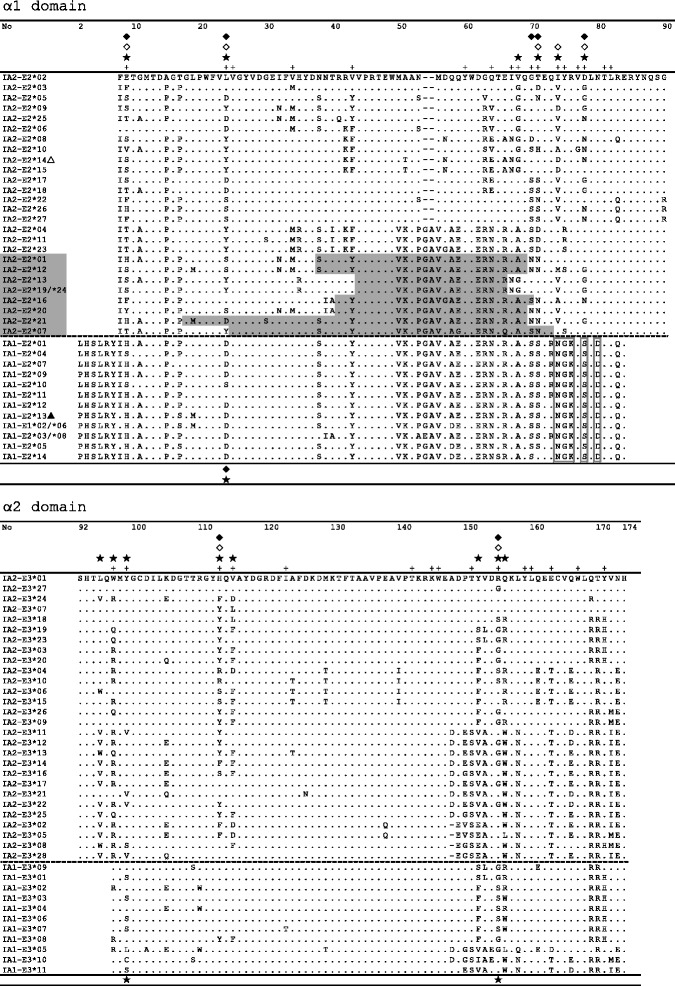
Golden pheasant *IA* (most of the α1 and α2 domains) amino acid alignment. Numbers above the alignment refer to residue positions. Locus-specific residues belonging to the *IA1* gene are indicated by open boxes. PBR sites predicted from consensus positions of chicken class I molecules [49, 50] are marked with a “+” symbol. The positively selected sites (PSSs) for *IA1* and *IA2* are indicated above and below the alignments, respectively. A filled asterisk represents PSS identified using the M8 site model, while a filled or open prism marks a PSS detected by REL or FEL, respectively. Interlocus recombination events determined by the RDP program produced 9 daughter recombinants, with their respective recombined regions indicated by grey shading; *IA2*-E2*14 as the major parent is denoted with an open triangle and *IA1*-E2*13 as the minor parent is denoted with a filled triangle

Our unpublished experimental data indicate that both *IA* genes are expressed in the golden pheasant. Through screening cDNA libraries using universal primers, 4 *IA* sequences were found for one pheasant; among them, 2 sequences corresponded to alleles from *IA1* and the other 2 were from *IA2*. Moreover, none of the putative amino acid sequences showed frameshift mutations for either gene, further implying that both genes were functional. For *IA1*, 16 of 83 (19 %) and 26 of 78 (33 %) amino acid sites were variable at exons 2 and 3. Fourteen alleles at exon 2 and 11 alleles at exon 3 coded for 12 and 10 distinct amino acid sequences, respectively (Fig. 2). Remarkably, the *IA1* alleles at exon 2 (*IA1*-E2) shared a locus-specific amino acid motif constituted by 5 conserved, discontinuous residues (N74, G75, K76, S78, and D80; Fig. 2). Such a striking feature, which resembled that reported in domestic chicken *BF1* alleles [38], might be used to distinguish *IA1* from *IA2*.

For *IA2*, 38 of 83 (46 %) and 27 of 83 (33 %) amino acid sites were variable at exons 2 and 3. Twenty-seven and 28 different amino acid sequences were encoded by the exon 2 alleles (−E2) and exon 3 alleles (−E3), respectively (Fig. 2). As mentioned above, the *IA2*-E2 alleles were the most variable and diverse and showed the greatest nucleotide and amino acid distances, especially in the PBR (Table 1), whereas the *IA1*-E2 alleles were the least diverse, with both distance values in the PBR slightly less than in the non-PBR.

**Table 1:** Nucleotide and amino acid distances of MHC I alleles for the golden pheasant

Locus	No. of alleles	K2P nucleotide distance			Poisson-corrected amino acid distance		
		All sites	PBR	Non-PBR	All sites	PBR	Non-PBR
*IA1*							
-E2 (254 bp)	14	2.9 (0.6)	2.1 (0.1)	3.1 (0.8)	6.6 (2.8)	4.4 (2.4)	7.4 (2.1)
-E3 (236 bp)	11	10.5 (1.6)	17.2 (5.4)	3.1 (0.8)	13.6 (2.8)	24.4 (8.9)	10.8 (2.9)
*IA2*							
-E2 (251 bp)	27	14.7 (1.9)	30.0 (7.5)	11.3 (1.7)	24.6 (4.1)	55.9 (21.9)	18.5 (3.7)
-E3 (253 bp)	28	14.7 (1.9)	30.0 (7.5)	11.3 (1.7)	24.6 (4.1)	55.9 (21.9)	18.5 (3.7)

Notes: Nucleotide distances were corrected for multiple substitutions with the Kimura 2-parameter model (K2P), and amino acid distances were corrected using expectations from the Poisson distribution. Distance values are given as percentages per site, with standard errors based on 1,000 bootstrap replicates (given in parentheses)

### Selection of 2 *IA* genes

Calculations for non-synonymous (*d*_N_: contributing to amino acid changes) and synonymous (*d*_S_: causing no amino acid change) substitutions for *IA1* and *IA2* are shown in Table 2. Regarding the putative PBR of *IA1*-E2, *d*_N_ was almost 4-fold higher than *d*_S_, although the difference between these 2 values was not statistically significant (Table 2). Non-PBR codons also yielded higher *d*_N_ than *d*_S_ values, but again the difference showed no significant deviation from neutrality (Table 2). Conversely, *IA2*-E2 alleles showed a significantly higher *d*_N_ than *d*_S_ in the PBR (*d*_N_/*d*_S_ = 9.444; Table 2), while *d*_N_/*d*_S_ was <1 for non-PBR codons. Surprisingly, for the α2 domain (E3) of both *IA* molecules, a lower *d*_N_ than *d*_S_ value was observed even in the PBR, although *d*_N_ in the PBR was approximately 2-fold higher than in the non-PBR.

**Table 2:** Calculations of non-synonymous (*d*_N_) and synonymous (*d*_S_) substitutions for golden pheasant MHC class I genes

Locus	All sites			PBR			Non-PBR		
	*d*_N_	*d*_S_	*d*_N_/*d*_S_	*d*_N_	*d*_S_	*d*_N_/*d*_S_	*d*_N_	*d*_S_	*d*_*N*_/*d*_S_
*IA1*									
-E2	3.3 (0.8)	1.7 (0.9)	1.94	3.5 (2.4)	0.9 (0.1)	3.89	3.3 (0.9)	2.0 (1.2)	1.65
-E3	7.7 (1.5)	10.6 (2.6)	0.72	12.1 (3.9)	14.1 (7.0)	0.72	6.2 (1.7)	9.6 (2.9)	0.65
*IA2*									
-E2	13.2 (2.4)	8.5 (2.3)	1.55	34 (9.6)	3.6 (1.8)	9.44^*^	8.7 (1.9)	10.5 (3.2)	0.83
-E3	10.4 (2.1)	15.0 (2.8)	0.69	19.1 (6.3)	28.8 (10.7)	0.66	7.9 (2.1)	11.4 (2.9)	0.69

Notes: The *d*_N_ and *d*_S_ were estimated using the modified Nei–Gojobori algorithm. Distance values are given as percentages per site, with standard errors based on 1,000 bootstrap replicates (given in parentheses). ^*^ *P* < 0.05, as determined by performing a 1-tailed Z test

Based on the alignment of 166 codons within the *IA2* gene, OmegaMap inferred 17 and 7 positively selected amino acid sites in exon 2 and exon 3, respectively. Fewer positively selected amino acids were found using other methods. Both the random-effects likelihood and fixed-effects likelihood (FEL) models identified 7 codons under positive selection (5 from exon 2, 2 from exon 3), while likelihood ratio tests, by comparison of M8 and M7 site models, detected 14 positively selected sites (6 from exon 2, 8 from exon 3; Table 3). Almost all sites under positive selection, as estimated using PAML software, were also detected using OmegaMap. Based on the combined results of the 4 methods, we concluded that 7 positive selection sites may be directly associated with peptide binding. However, there were fewer codons in the *IA1* gene under positive selection (10, 3, 1, and 0 codons found using the M8 vs. M7 in OmegaMap, PAML, REL, and FEL models, respectively; Table 3).

**Table 3:** Inference of positively selected amino acid sites for golden pheasant MHC class I sequences

Locus	M8 vs. M7 likelihood ratio test			Positively selected sites			
	df	Test statistic	Significance	OmegaMap	PAML	REL	FEL
*IA1*							
-E2	2	6.7	<0.01	24 L *	24 L **	24 L **	-
-E3	2	10.5	<0.001	(9^A^)	99Y**, 155R**	-	-
*IA2*							
-E2	2	105.8	<0.001	(17^B^)	9E**, 24 L**, 68 V**,	9E**, 24 L**,	9E**, 24 L**,
					71 T**, 74I**, 78D**	70G**, 71 T**, 78D**	71 T**, 74I*, 78D*
-E3	2	47.3	<0.001	(7^C^)	95 L**, 97 W**, 99Y**, 113H**,	113H**, 155R**	113H**, 155R*
					115 V**, 152Y*, 155R**, 156Q*		

Notes: The test statistic was computed as 2 (Lb-La), where La and Lb are log-likelihood values for the M8 and M7 site model, respectively. *, posterior probability > 0.95; **, posterior probability > 0.99. df, degree of freedom; PAML, phylogenetic analysis by maximum likelihood; REL, random effects likelihood; and FEL, fixed-effects likelihood. ^A^ The inferred positively selected amino acid sites by OmegaMap in *IA1*-E3 were: 99Y*,150P**, 151 T**, 152Y**, 153 V**, 154D**, 155R**, 156Q** and 158 L**. ^B^ The inferred positively selected amino acid sites by OmegaMap in *IA2*-E2 were: 9H**, 24 L**, 40 T*, 41R*, 42R*, 55A**, 56 M*, 57D**, 58Q**, 59Q*, 67I**, 68 V**, 70G**, 71 T**, 74I**, 75Y**, and 78D**. ^C^ The inferred positively selected amino acid sites by OmegaMap in *IA2*-E2 were: 113H**, 115 V*, 150P**, 151 T*, 152Y**, 155R**, and 156Q**

### Phylogenetic analysis

**Fig. 3:**
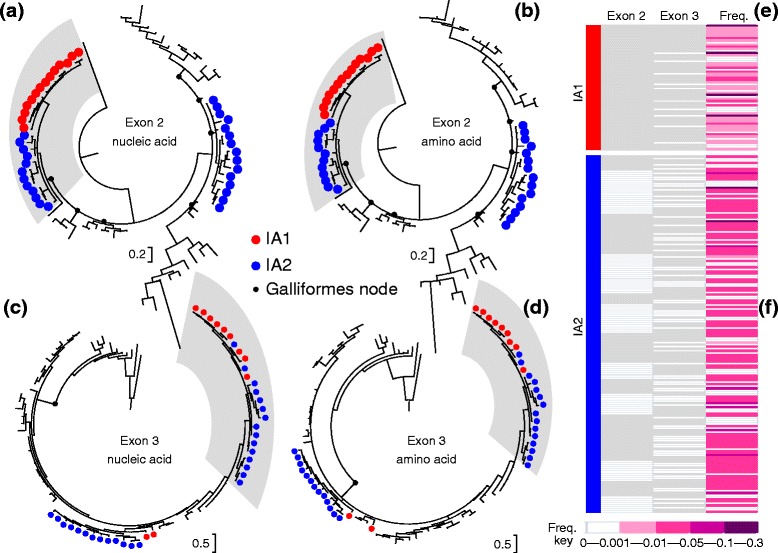
Bayesian trees of *IA* *1*-E2 nucleic (**a**) and amino (**b**) acids, *IA2*-E3 nucleic (**c**) and amino (**d**) acids, and the recombination between exons 2 and 3 in *IA1* (**e**) and *IA2* (**f**). The NJ tree was not shown, but was similar to the Bayesian trees. The sequences used to generate the trees are as follows: *Gallus gallus*: *Gaga*-BF1*01 (Z54314.1), *Gaga*-BF1*02 (Z54318.1), *Gaga*-BF1*03 (Z54320.1), *Gaga*-BF1*04 (Z54322.1), *Gaga*-BF1*05, *Gaga*-BF2*01 (Z54315.1), *Gaga*-BF2*02 (Z54316.1), *Gaga*-BF2*03 (Z54317.1), *Gaga*-BF2*04 (Z54319.1), *Gaga*-BF2*05 (Z54321.1); *Meleagris gallopavo*: *Mega*-Ia1*01 (FJ917378.1), *Mega*-Ia1*02 (FJ917382.1), *Mega*-Ia1*03 (FJ917384.1), *Mega*-Ia1*04 (FJ917380.1), *Mega*-Ia1*05 (DQ993255.2), *Mega*-Ia2*01 (DQ993255.2), *Mega*-Ia2*02 (FJ917381.1), *Mega*-Ia2*03 (FJ917379.1), *Mega*-Ia2*04 (FJ917383.1); *Coturnix japonica*: *Coja*-*B1* (AB005529.2), *Coja*-*B2* (AB005530.1), *Coja*-*C* (AB005527.1), *Coja*-*D1* (AB078884.1), *Coja*-*D2* (AB078884.1), *Coja*-*E* (AB078884.1); *Tetrao tetrix*: *Tete*-*BF1* (JQ028669.1), *Tete*-*BF2* (JQ028669.1); *Tympanuchus cupido*: *Tycu*-*IA*-E3*01-*14 (JX237356-69), *Tycu*-*IA* (JX971120.1); *Numida meleagris*: *Nume*-BF2*01 (EU430728.1), *Nume*-BF2*02 (EF643463.1); *Acrocephalus arundinaceus*: *Acar*-MHC-I (AJ005503.1); *Larus scopulinus*: *Lasc*-*UAA* (HM015819.1), *Lasc*-*UBA* (HM015820.1), *Lasc*-*UCA* (HM015821.1), *Lasc*-*UDA* (HM008716.1); *Calidris canutus*: *Caca*-*UA* (KC205140.1); *Anas platyrhynchos*: *Anpl*-*UAA* (AY885227.1), *Anpl*-*UBA* (AY885227.1); *Anser anser*: *Anan*-MHC-I (AY387652.1). *Sphenodon punctatus* sequence: *Sppu*-*U* (DQ145789.1) was used to root the tree

The golden pheasant MHC class I sequences clearly clustered with published MHC I sequences from other Galliformes species, but they were separated from those of non-Galliformes birds both in the nucleic acid (Fig. 3a and c) and amino acid (Fig. 3b and d) phylogenetic trees. Within the Galliformes clade, sequences belonging to the same species tended to group together. In Fig. 3a and b, all *IA1*-E2 alleles and 12 *IA2*-E2 alleles of the golden pheasants formed an independent clade, while all *IA2*-E2 alleles fell into 2 relatively well-distinguished clusters due to extreme sequence variability. Similarly, those E3 alleles, which displayed great sequence divergences characteristic of the classical class I genes, mostly fell into 2 major clusters except for *IA1*-E3*05 and *IA1*-E3*10 (Fig. 3c and d); within one cluster, alleles from both loci comingled with each other. In addition, the network relationship among class I sequences of 7 Galliformes species also signified the absence of orthologous relationships (Additional file 1: Figure S1).

### Recombination of MHC I loci in the golden pheasant

The alignment results between *IA1* and *IA2* revealed that the intermediate 44–66 amino acid residues of alleles *IA2*-E2*01, 04, 07, 11–13, 16, 19, 20, 21, 23, and 24 were nearly equivalent to the corresponding region of alleles *IA1*-E2*01, 04, 07, 09–11, and 13 (in Fig. 2), which was suggestive of intergenic recombination. This pattern was subsequently verified using at least 3 methods in the RDP software program. Those interlocus recombination events occurred intensively between *IA2*-E2*14 as the major parent (marked with △ in Fig. 2) and *IA1*-E2*13 as the minor parent (marked with ▲ in Fig. 2). They ultimately produced 9 daughter recombinants (their respective recombined regions are shown by grey shading in Fig. 2), accounting for a substantial proportion (33.3 %) of the *IA2*-E2 alleles. Thus, a genomic region (31–190 nt) of *IA2*-E2*16 was replaced respectively by its counterpart in *IA1*-E2*13 during the recombination process. Accordingly, one significant breakpoint at 109 nt (*P* < 0.01, after sequential Bonferroni correction) was detected using the GARD program. We also assessed recombination between exons 2 and 3 in *IA1* (Fig. 3e) and *IA2* (Fig. 3f), respectively. The bars (PHASE-based haplotypes) with fore-grey and after-white were the products of recombination between exon 2 (grey clusters; Fig. 3a and b) and exon 3 (white or blank clusters; Fig. 3c and d), and vice versa for the fore-white after-grey bars. Among these bars, those with a frequency higher than 0.1 should reflect inter-exon recombination hotspots.

### Genetic diversity and population-structure analysis of MHC I loci across golden pheasant populations

Within the populations studied, the number of *IA1* alleles ranged from 5–11 at exon 2 and from 3–9 at exon 3, whereas the number of *IA2* alleles ranged between 6 and 22 at either exon 2 or exon 3 (Additional file 2: Table S1). To handle the variation of sample sizes across the different populations, allelic richness was standardized based on a minimal sample size of 9 diploid individuals, using the rarefaction method. In this case, estimates of the allelic richness at each locus still differed among the populations (*IA1*-E2: 4.4–6.7; *IA1*-E3: 3–5.7; *IA2*-E2: 5.9–8.8; and *IA2*-E3: 5.9–9.5; Additional file 3: Table S2). Five *IA1* alleles (*IA1*-E2*03, 06 and *IA1*-E3*01, 02, 05) and 6 *IA2* alleles (*IA2*-E2*03, 04 and *IA2*-E3*01, 03, 08, 18) were observed throughout all the populations studied. In particular, 8 alleles (*IA1*-E2*03, 06, and *IA1*-E3*02, 05; *IA2*-E2*03, 04; and *IA2*-E3*01, 03) were most common, although the allele frequencies varied among populations (Additional file 2: Table S1). For *IA1*, most populations exhibited less heterozygosity than expected, and all instances conformed to the Hardy–Weinberg equilibrium (HWE) after Bonferroni correction (Additional file 3: Table S2). For *IA2*, a similar pattern was observed except for 3 cases with significant heterozygote deficits (*IA2*-E2 in Hunan [HN] and *IA2*-E3 in Changqing [CQ] and Lichuan [LC]; Additional file 3: Table S2). We inferred that the presence of null alleles may not be the main cause of HWE departures in these 3 cases because their frequencies were all <0.1, as estimated by the expectation-maximization algorithm.

**Fig. 4:**
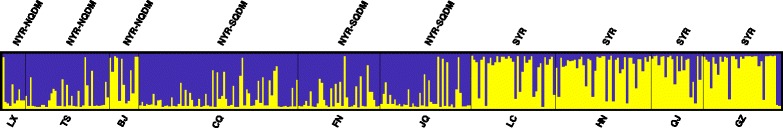
Structure clustering results obtained for K = 2. Each individual sample is represented by a thin vertical bar, and the length of each bar corresponds to the posterior probability within each cluster. Populations are separated by black bars, with the names indicated below the graph and the specific geographic locations marked above the graph. NYR, the northern region of the Yangtze River; SYR, the southern region of the Yangtze River; NQDM, northern areas of Qinling-Daba Mountain; SQDM, southern areas of Qinling-Daba Mountain. LX, Linxia; TS, Tianshui; BJ, Baoji; CQ, Changqing; FN, Foping-Ningshan; JQ, Jiange-Qingchuan; LC, Lichuan; HN: Hunan; QJ: Qianjiang; GZ, Guizhou. K, the number of putative clusters

The genetic clustering analysis, computed with a non-linkage model in STRUCTURE, revealed that ΔK reached a peak at K = 2 (mean values: LnP (D) = −5272.2; Additional file 4: Figure S2), indicating that the split in the dataset divided the populations into 2 major genetic clusters. The blue cluster (Fig. 4) is dominantly composed of populations scattered across the northern region of the Yangtze River (NYR; including Linxia [LX], Tianshui [TS], CQ, Foping-Ningshan [FN], and Jiange-Qingchuan [JQ]), except for Baoji (BJ). The yellow cluster mainly represents populations located in the southern region of the Yangtze River (SYR; consisting of LC, Qianjiang [QJ], HN, and Guizhou [GZ]; Fig. 4). Based on clustering results, 10 populations were separated into 2 groups for subsequent analysis: NYR (LX, BJ, TS, CQ, FN, and JQ) and SYR (LC, QJ, HN, and GZ). BJ appeared slightly distant from the other NYR populations, probably because of the lack of representative samples. We also performed analysis with a linkage model in STRUCTURE software. The same ΔK and grouping were obtained, but the relative proportion of each population in either cluster was different. The association between the SYR and NYR groups was clearly demonstrated in both non-linkage and linkage models.

A congruent pattern from STRUCTURE analysis was also obtained when genetic differentiation among populations over the MHC class I loci was estimated by *F*_*ST*_ (Additional file 5: Table S3). After omitting the BJ population, pairwise *F*_*ST*_ values exhibited no significant differentiation within the NYR or SYR populations (NYR: *F*_*ST*_ range 0.001–0.030, *P* > 0.01, mean *F*_*ST*_ = 0.016; SYR: *F*_*ST*_ range 0.009–0.059, *P* > 0.01, mean *F*_*ST*_ = 0.036), whereas pairwise *F*_*ST*_ statistics showed significant differentiation between NYR and SYR populations (*F*_*ST*_ range 0.019–0.129, *P* < 0.01; mean *F*_*ST*_ = 0.069), suggesting that all populations, except BJ, tended to form 2 major groups (NYR [LX, TS, CQ, FN, JQ] and SYR [LC, QJ, HN, GZ]) that were divided by the Yangtze River. A Mantel test showed that the observed correlation between pairwise *F*_*ST*_ values and geographical distances was significantly different from zero (r = 0.389, *P* = 0.013 < 0.05), indicating that a strong isolation-by-distance relationship occurred for MHC class I variations.

During analysis of molecular variance (AMOVA), we examined the genetic structure pattern of the 2 groups, after completely excluding BJ. The 2-group analysis indicated that 93.65 % of the genetic variation was distributed within populations, 4.07 % of the total variance was partitioned between groups, and 2.28 % occurred among populations within groups (Table 4). The results demonstrated that the genetic variation between groups (Va) was significantly greater than the genetic variation among populations (Vb) (Table 4), suggesting that the Yangtze River might serve as a barrier impeding gene flow between the NYR and SYR populations.

**Table 4:** Summary of AMOVA results for MHC class I loci from golden pheasant populations

Source of variation	df	SS	Variance components	Percentage of variation	Fixation index	*P*
Between groups	1	26.77	Va: 0.07	4.07	F 0.041	<0.00001
Among populations within groups	7	30.08	Vb: 0.04	2.28	F_SC_ 0.024	<0.00001
Within populations	649	1030.58	Vc: 1.59	93.65	F_ST_ 0.064	<0.00001

Notes: Significance tests are based on 1,023 permutations. AMOVA, analysis of molecular variance; df, degree of freedom; SS, sum of squares of deviations from mean

## Discussion

### Extensive allelic variation in MHC class I genes

We characterized, for the first time, the second and third exons of MHC class I genes in 12 wild populations of golden pheasant. We identified alleles for the *IA1* locus (14 E2 alleles and 11 E3 alleles) and for the *IA2* locus (27 E2 alleles and 28 E3 alleles), which differed markedly in their levels of allelic divergence. *IA2* displayed more extensive sequence variations, perhaps because of differential selection pressure or as a reflection of its inherently different properties. Forty-one different alleles in the second exon and 39 different alleles in the third exon were found among 339 individuals, revealing a high polymorphism of 2 peptide-binding domains in the golden pheasant class I genes. In previous studies, other non-model avian species also exhibited extraordinary diversity at exon 3 of MHC class I genes. For example, 36 MHC class I alleles were identified in a screening of 8 red knots [39], 50 unique functional class I variants were detected in a natural population of blue tits [40], and up to 82 divergent class I gene sequences were observed in the scarlet rosefinch population [6]. However, class I sequences from these avian species were generally amplified using species- or motif-specific PCR, which does not involve assignment to particular loci. Hence, for these studies, it was not possible to explore the evolutionary processes underlying MHC variation at each locus.

### Differential MHC polymorphism driven by differential selection pressure

The main force generating and maintaining MHC polymorphism appears to be balancing selection [41, 42]. Nucleotide sites under positive selection are expected to accumulate more non-synonymous than synonymous substitutions, eventually bringing about amino acid changes and corresponding functional changes in MHC proteins [43]. Such adaptive evolutionary processes due to parasite-driven selection should be evident at the PBR [44]. According to the proposed criteria, both *IA* loci of the golden pheasant showed evidence of positive selection for diversification. For *IA2*-E2, positive selection acted only on PBR codons, which showed a significantly increased degree of non-synonymous substitutions (*d*_N_/*d*_S_ = 9.44, *P* < 0.001). *IA1*-E2 alleles also evolved under positive selection, although not significantly. E2 alleles of both *IA* genes exhibited higher *d*_N_/*d*_S_ ratios at PBR codons than at non-PBR codons, which agrees with the hallmark of such functionally important regions as targets of balancing selection [45]. However, compared with *IA1*, the *IA2* gene showed more allele polymorphisms and more frequent nucleotide substitutions, implying that increased selection pressure more effectively increased the functional variation at *IA2*. Though no significant recombination events were detected in E3 sequences, interlocus homogenization implied that these 2 loci might have evolved by concerted evolution. Previous findings have confirmed that different patterns of allele polymorphisms occurring at MHC genes are subject to the divergence of selection intensities between loci. In humans, the intensity of selection appears to have differed among 3 *HLA* class I loci; *HLA*-*B*, which was affected by the strongest selection, is more polymorphic than the other 2 class I loci, *HLA*-*A* and *HLA*-*C* [46]. In the alpine newt, increased variation in the *DAB* gene, composed of 37 alleles, was largely caused by strong selection over an evolutionary timescale, while low variation in the *DBB* gene, containing only 3 alleles, was correlated with a lack of selection pressure [47]. In contrast, the lack of increased *d*_N_ over *d*_S_ in the PBR of E3 alleles indicates that positive selection might be less advantageous in the α2 domain. Stronger selection in the α1 domains than in the α2 domains could be ascribed to structural principles, which crucially govern the important peptide-binding motifs of class I molecules [48]. In addition, without exact knowledge of the crystal structures of class I molecules, the putative golden pheasant PBR deduced from consensus sites of chicken [49, 50], might vary considerably from the actual PBR of golden pheasant class I proteins, especially for the α2 domains. However, *d*_N_ in the PBR at exon 3, which was twice that determined for the non-PBR, suggests that selection might also have played a role in maintaining their variation.

Based on the predictive outcomes of 4 codon-based models, we found substantial evidence of balancing selection at individual codons, as well as divergent selective pressure between *IA1* and *IA2*. Seven positively selected sites were identified in *IA2* by at least 2 codon-based models (Fig. 2; Table 3), and all of them corresponded to peptide-recognition sites in chickens [49, 50]. A considerable amount of non-synonymous variation occurred at those specific codons under positive selection (Fig. 2), demonstrating that polymorphisms within the α1 and α2 domains may be of functional importance [4]. Conversely, fewer positively selected sites were detected in *IA1*. Therefore, selective pressure on both *IA* loci differed strikingly, and *IA2* underwent much stronger positive selection, especially in the PBR. In conclusion, differing levels of diversity and differential selection might be indicative of different functional roles for the golden pheasant *IA* loci.

### Phylogeny and interlocus recombination

The pattern of MHC evolution in birds appears to be quite different from that described in mammals [7, 51–53]. Mammalian MHC genes independently evolved, as reflected by phylogenetic trees showing that corresponding orthologous sequences from different species grouped more closely than other sequences from the same species [25]. In birds, the lack of orthologous relationships among putatively different MHC loci is common, and such examples of phylogeny-based orthology in MHC genes are limited to pairs of closely related avian species [54–57]. However, the phylogenetic inconsistency in avian species is mainly due to higher frequencies of interlocus recombination, also termed “concerted evolution” [51–53, 58]. Frequent recombination events occurring in MHC multigene families usually produce between-locus homogenization, which obscures the true phylogenetic relationship. Similar to some cases observed in class II genes in Galliformes [35, 57, 59], golden pheasant class I sequences failed to constitute well-supported independent groups in accordance with each gene. Instead, class I sequences appeared to group together and were intermixed (Fig. 3), which is consistent with concerted changes. Thus, the occurrence of intergenic recombination between *IA1* and *IA2* was discernibly responsible for the locus-intermingling pattern, underpinning the diversification of MHC genes in the golden pheasant.

### Population structure based on MHC class I variation

The *IA1* and *IA2* genes both exhibited a large variation in allele numbers across sampled populations, i.e., 6–22 alleles were found at *IA2*-E2 in each population (Additional file 2: Table S1), as well as in terms of sample size-corrected allelic richness. Alternatively, a large difference in the number of alleles with a frequency of ≤0.05 was found among populations, i.e., 0–17 at *IA2*-E2 and 5–19 at *IA3*-E3 (Additional file 2: Table S1), indicating that rare alleles have been retained in the golden pheasant populations by frequency-dependent selection [15], a potential mechanism accounting for the extreme polymorphism and long-term persistence of alleles at the MHC. Furthermore, the widespread sharing of class I alleles (i.e., *IA2*-E2*03 and *04) across all populations suggests that these alleles may have predominated in golden pheasant populations in recent times. If the same dominant pathogen widely invades different golden pheasant populations, a class I allele that confers a selective advantage in resistance against this prevalent pathogen is quite likely to be shared among populations.

The comparison of genetic differentiation between populations at various spatial scales also supports the hypothesis of balancing selection, which is generally regarded as the main force shaping MHC genetic diversity [14, 42]. In a relatively small geographic region, where populations experience very similar pathogen-mediated selective regimes, i.e., homogenous selection pressure, balancing selection tends to decrease the levels of between-population variation compared to within-population variation [41, 60, 61]. Therefore, the little differentiation among golden pheasant populations from the NYR or SYR might be a result of homogenous balancing selection. However, the structuring of genetic variation at MHC loci indicated that the Yangtze River itself shaped the population structure of golden pheasants. The Yangtze River, as the largest river in China, is too wide for golden pheasants to cross because they do not participate in long-distance migratory flight. Hence, the Yangtze River, an important natural barrier to dispersal and gene flow for golden pheasant populations, intensified the genetic divergence and eventually caused the formation of 2 distinctive population groups: NYR and SYR. Significant inter-population differentiation between the NYR and SYR clusters probably also resulted from geographic heterogeneity of balancing selection between distant populations, which reportedly occurs because of variation in pathogen communities at a broad geographic scale [60, 62–64].

## Conclusion

In the present study, we genotyped MHC class I genes from 12 wild golden pheasant populations by PCR-SSCP, using locus-specific primers. Our work revealed that: 1) 2 MHC class I genes exhibited differential genetic polymorphisms, and much stronger positive selection detected at *IA2* than at *IA1* might account for the more extensive variation of *IA2*; 2) interlocus recombination between 2 *IA* genes, noticeably reflected by the intermingling phylogenetic pattern, is also an important mechanism responsible for the extensive allelic variation of the *IA2* gene; 3) the pattern of population differentiation implied that homogenous balancing selection might explain why an even distribution of MHC variation was maintained among populations within the NYR or SYR region, while the Yangtze River acted as a barrier to gene flow between NYR and SYR populations, and heterogeneous balancing selection might be an important factor determining the NYR-SYR genetic structure in golden pheasants.

## Methods

### Ethics statement

Muscle tissues were collected from deceased pheasants obtained from poachers and pheasants that died from natural causes, found at nature reserves (NRs). No pheasants were killed specifically for this study. Blood samples were obtained under standard veterinary care during physical examination of rescued birds, i.e., blood samples were not taken solely for the purpose of this study. We collected muscle and blood samples as gene resources with permission from the Department of Wildlife Conservation and Nature Reserve Management under the State Forestry Administration of China, and deposited them (Chpi0001–Chpi0339) in the State Conservation Center for Gene Resources of Endangered Wildlife of China. The pheasant samples used in this study were from LX Taizishan National NR, TS Maicaogou NR, BJ Shenshahe NR, CQ National NR, FP National NR, NS NR, JG Xihe NR, QC Tangjiahe National NR, LC Xingdoushan National NR, Sangzhi (SZ) Badagongshan National NR, Yongshun (YS) Xiaoxi National NR, Jishou (JS) Baxianhu NR, QJ Wulingshan NR, Jiangkou (JK) Fanjingshan NR, Shibing (SB) Fodingshan NR, and Libo (LB) Maolan National NR. We did not collect samples from any other species at these NRs.

### Sample collections from Chinese golden pheasants

We collected 339 golden pheasant muscle and blood samples from the above-mentioned 16 populations throughout Mainland China, of which few samples were collected from pheasants in SZ, YS, and JS (HN Province) or in JK, SB, and LB (GZ Province); thus, the intra-province samples were incorporated into the HN and GZ populations, respectively. Among these 12 populations, 8 populations were distributed within the NYR (3 were scattered among the northern range of the QDM [NQDM]), and the other 5 were located near the southern foothills of the QDM [SQDM]), whereas 4 populations were distributed within the SYR (Fig. 1). Sample sizes of the NS and QC populations were relatively small (7 samples from the NS region and 8 from the QC region); therefore, for convenience in discussing the population structure, we integrated the NS population with the adjacent FP population (i.e., designated as FP-NS or FN) and the QC population with JG (i.e., designated as JG-QC or JQ). Sample collection proceeded as follows: blood (approximately 100 μL for each living pheasant) was collected by brachial venipuncture, while muscle tissues were obtained from dead pheasants. Genomic DNA was phenol-chloroform extracted, as described previously [65].

### Locus-specific amplifications

Based on direct evidence of cDNA expression in one pheasant (our unpublished experimental data), the 2 MHC class I genes (*IA1* and *IA2*) are expressed. To comprehensively characterize variation in the highly variable regions (exons 2 and 3) in *IA1* and *IA2*, we developed locus-specific screening methods based on knowledge of the MHC genomic structure in the golden pheasant [37]. The locus-specific primer set (A-E2-up1/A-E2-dn1) was designed to amplify fragments of 346 bp in length, which encompassed most of exon 2 in *IA1*. A 10-μL polymerase chain reaction (PCR) mixture was prepared, which included 20 ng of extracted DNA, 5 μL of 2 × GC I Buffer with MgCl_2_ (TaKaRa, China), 0.2 μM primers (synthesized by Invitrogen, USA), 0.2 mM dNTPs (TaKaRa, China), and 0.25 U Ex-Taq Polymerase (TaKaRa). The PCR conditions were as follows: 95 °C for 5 min; 30 cycles of 95 °C for 30 s, 55 °C for 30 s, and 72 °C for 30 s; followed by 72 °C for 10 min. Nested PCR was used to specifically amplify exon 3 of the *IA1* gene. A pair of primers (A-E3-e2up1/A-E3-i3dn1) was used during the first round of amplification, and then a pair of internal primers (A-E3-e2up1/A-E3-e3dn2) was used to amplify a shorter target fragment (272 bp), which was appropriate for subsequent genotype screening. Exon 3 of *IA1* was amplified by nested PCR based on the reaction scheme used to amplify exon 2, except that the cycle number was decreased to 20 in the first round of amplification.

The complete *IA2* gene was amplified with the primers TAP1-E11-upper22 and C4-E1-down11, which targeted long fragments of approximately 4.2 kb spanning from the *TAP1* gene to the *C4* genes [37]. PCR was performed using LA-Taq Polymerase (TaKaRa), and the thermocycling conditions were as follows: an initial step of 94 °C denaturation for 1 min; 18 cycles of 98 °C for 10 s and 68 °C for 5 min; and 72 °C for 10 min. Then, using the *IA2* amplicons as templates, 2 other pairs of primers (*Ch.pi*-I1up3/*Ch.p*i-I2dn1 and *Ch.pi*-I2up5/A-E3-e3dn2) were employed in nested-PCRs to amplify exons 2 or 3 of *IA2*, using similar reaction conditions and protocols as described for *IA1*. Consequently, the final products contained only the *IA2* locus. The positions and sequences of all primers used in this study are shown in Figure S3 of Additional file 6.

### SSCP genotyping and allele nomenclature

SSCP is a highly sensitive, rapid, convenient, and relatively inexpensive technique for genotyping. By discriminating subtle differences in shifted SSCP bands among different DNA sequences, this method easily detects 1- or 2-nucleotide mutations [66, 67]. Hence, we combined SSCP with DNA sequencing to survey for MHC variations, an approach that has been broadly applied for studying various species [5, 31, 68].

SSCP was performed using a DCode Universal Mutation Detection System (Bio-Rad, USA), according to the manufacturer’s instructions and the detailed description by Wan QH et al. [68]. Briefly, 5 μL of each PCR product was diluted 1:1 in loading dye. The mixture was denatured for 5 min, immediately placed on ice to halt further reaction, and loaded onto a non-denaturing polyacrylamide gel (8–12 % acrylamide). Electrophoresis was conducted at a constant running temperature of 8 °C and at 30 W for 8–12 h. SSCP bands were visualized by silver staining [69]. PCR product bands were excised from the gels, and allele sequences were determined by directly sequencing the PCR products. TA cloning and sequencing were also performed to identify novel MHC alleles. We purified the source PCR products using the AxyPrep™ DNA Gel Extraction Kit (Axygen Biosciences, USA), inserted them into the pMD18-T vector (TaKaRa), and transformed the recombinant plasmids into DH5α competent cells (TaKaRa). In general, at least 3 positive clones per allele were picked for sequencing to confirm their authenticity. To avoid PCR artifacts, all alleles identified in this study were verified at least in 2 different pheasant samples when possible, and alleles found only in one pheasant were validated by performing 3 independent PCRs.

Each single allele was assigned an individual name, consisting of the specific locus (*IA1* or *IA2*), the specific exon (e.g., E2, exon 2), and a sequential number following an asterisk (starting from 01), referring to the nomenclature used for chicken MHC [54]. All *IA* alleles can be accessed in GenBank under the following Accession Numbers: *Chpi*-*IA1*-E2*01–*14 (KM005661–89), *Chpi*-*IA1*-E3*01–*11 (KM005650–60), *Chpi*-*IA2*-E2*01–*27 (KM005703–29), *Chpi*-*IA2*-E3*01–*28 (KM005675–702).

### Sequence analysis

Nucleotide sequence alignments and the corresponding amino acid translations were performed using MEGA5 [70]. Estimations of mean pairwise nucleotide distances and amino acid distances were determined by MEGA5. We then employed the modified Nei–Gojobori algorithm [71] via the Jukes-Cantor correction [72] using MEGA5 to compute *d*_N_ and *d*_S_. Standard errors were determined as bootstrap values with 1,000 replicates.

The *d*_N_/*d*_S_ ratios were evaluated for PBRs, non-PBRs, and all sites of the second and third exons in both investigated genes. Significances were determined by a 1-tailed Z test in MEGA5. The putative peptide-binding residues were inferred from codons corresponding to PBRs of chicken MHC class I molecules [49, 50]. The comparison of codon-based substitution models of sequence evolution for the *IA1* and *IA2* genes was accomplished by 2 methods. First, selections were evaluated using OmegaMap [73]. Without prior knowledge, all codons were assumed to have an equal equilibrium frequency and all priors were set as recommended by Wilson & McVean [73], with the exception of ω and ρ models, which were allowed to independently vary across codons. We ran 500,000 Markov chain Monte–Carlo iterations with a thinning interval of 100. Second, a maximum-likelihood approach in PAML 4.2 [74] was also taken. For simplicity, the nested site models (M7 and M8), which assume ω variation (ω = *d*_N_/*d*_S_) with a beta distribution among sites, were used for our study. In the likelihood-ratio test [75], the pairing models were compared with each other. The M8 model with an extra 11 class sites, allowed the identification of positively selective sites from the Bayes empirical Bayes inference [76], and only sites with posterior probabilities higher than 95 % were considered to be under positive selection. However, for recombination sites, the likelihood methods tend to overestimate ω values among sites and might produce high false positive rates for positively selected sites [77]. Therefore, based on non-recombinant sequences only (see below), 2 additional analyses were applied to infer selection with respect to particular codons using a Bayesian approach: REL [54] assumed the substitution rates across sites under a fixed distribution and then made inferences about the ω rate at each site, while FEL directly estimated ω values for individual sites without any assumption. Both methods were implemented by HyPhy software [78] and are available at the Datamonkey web interface (http://www.datamonkey.org/) [79].

Phylogenetic reconstructions using golden pheasant MHC I alleles determined herein and other avian sequences available in the National Center for Biotechnology Information (NCBI) were performed separately for exons 2 and 3. The corresponding class I sequence from tuatara (*Sphenodon punctatus*) was used as an outgroup. A Bayesian phylogeny was reconstructed using MrBayes software, version 3.2 [80] with the posterior probabilities inferred from Metropolis-coupled Markov chain Monte Carlo simulations (MCMCMCs). Two independent MCMCMC simulations (4 chains each, temperatures set at 0.20) were run for 10,000,000 generations with tree sampling every 1,000 generations for both genes until reaching convergence. The first 25,000 sampled trees were excluded as burn-in. The remaining trees were used to construct the 50 % consensus tree using the GTR (for nucleotide) and JTT (amino acid) models, and the resulting trees were displayed in TreeView [81]. Neighbor-joining (NJ) trees were generated by MEGA5 [82] for comparison with the Bayesian trees. A bootstrap test was performed to estimate support for the nodes in the tree via 1,000 replicates. In addition, Neighbor–Net networks of golden pheasant alleles, together with class I sequences of Galliformes birds, were established using Kimura’s 2-parameter model in Splits Tree 4 [83].

RDR4 software [84] was used with the default setting to detect recombination. Recombination analyses were conducted by at least 3 RDR4-implemented algorithms, including RDP [85], GENECONV [86], and MAXCHI [87]; only recombination events that were well verified by all these methods were considered valid. Moreover, we examined the possibility of recombination using the GARD algorithm [88] from the Datamonkey webserver (http://www.datamonkey.org/). The haplotypes between exons 2 and 3, as well as inter-exon recombination events were both computed by PHASE version 2.1.1 (http://stephenslab.uchicago.edu/software.html#phase).

MHC allele frequencies, number of alleles, allelic richness, *H*_*O*_ and *H*_*E*_ were calculated using FSTAT software version 2.9.3 [89]. The rarefaction method [90, 91] was utilized to correct differences in sample sizes across populations during the measurement of allelic richness. The extent of deviation from HWE was evaluated by Monte Carlo simulation tests with sequential Bonferroni correction [92] in GenePop 4.0 [93]. Based on multilocus genotype data from the studied populations, we inferred the genetic structure by employing the Bayesian clustering algorithm in STRUCTURE V 2.3.3 [94]. We employed non-linkage and linkage models to infer population structures. Typically, the number of putative clusters (K) was assumed to be no more than the number of actual sampled populations; hence, the K value ranged from 1–10. For each K value, 10 independent runs were performed using default settings to obtain log likelihoods estimated by Ln Prob, mean value of ln likelihood, and variance of ln likelihood [95]. The most probable value of K was eventually determined by the model choice criterion ΔK [95], which was dependent on the second-order change of the log likelihood mean. The outputs from the Bayesian analyses were visualized with DISTRUCT v1.1 [96]. Pairwise *F*_*ST*_, as an index of population differentiation, was estimated with the Weir and Cockerham method [97] in Arlequin3.5 [98], and the significance levels of *F*_*ST*_ values were evaluated by performing 10,000 permutations [99, 100]. Mantel tests were conducted for the detection of isolation by distances at MHC class I genes. First, geographical distances between populations were measured using Google Earth [101]. Then, the correlation between the natural logarithm of the geographical distance and *F*_*ST*_/(1-*F*_*ST*_) for MHC was tested using a simple mantel test in ZT [102] with 10,000 randomizations. AMOVA tests were performed using Arlequin3.5 software [98] to better partition the genetic variation between groups (Va), among populations (Vb), and within populations (Vc).

### Availability of supporting data

The data set supporting the results of this article is available in the LabArchives [DOI: 10.6070/H4RR1W8K].

## Additional files

Additional file 1: Figure S1. Network tree of exons 2 (A) and 3 (B) for golden pheasant MHC class I genes. (PDF 4 MB) Additional file 2: Table S1. Allele frequencies at MHC class I genes across golden pheasant populations. (PDF 611 kb) Additional file 3: Table S2. Summary of MHC genetic diversity in golden pheasant populations. (PDF 365 kb) Additional file 4: Figure S2. Results of Bayesian clustering analysis performed in STRUCTURE. (PDF 166 kb) Additional file 5: Table S3. Pairwise *F*_*ST*_ values among populations in MHC class I genes. (PDF 261 kb) Additional file 6: Figure S3. Locus-specific primers amplifying exons 2 and 3 of golden pheasant MHC class I genes. (PDF 2 MB)

## Abbreviations

AMOVA: analysis of molecular variance
BJ: Baoji
BS: bootstrap support
CQ: Changqing
*d*_N_: non-synonymous substitutions
*d*_S_: synonymous substitutions
FEL: fixed effects likelihood
FN: Foping-Ningshan
FP: Foping
*F*_*ST*_: F-statistic
GTR: general time reversible
GZ: Guizhou
*H*_E_: expected heterozygosity
HN: Hunan
*H*_O_: observed heterozygosity
HWE: Hardy-Weinberg equilibrium
JG: Jiange
JK: Jiangkou
JQ: Jiange-Qingchuan
JS: Jishou
JTT: jones-taylor-thornton
LB: Libo
LC: Lichuan
LX: Linxia
MCMCMC: metropolis-coupled Markov chain Monte Carlo simulations
MHC: major histocompatibility complex
NCBI: national center for biotechnology information
NJ: neighbor-joining
NQDM: Northern range of Qinling-Daba Mountain
NS: Ningshan
NYR: the northern region of the Yangtze River
PBR: peptide-binding region
QC: Qingchuan
QDM: Qinling-Daba mountain
QJ: Qianjiang
REL: random effects likelihood
SB: Shibing
SQDM: Southern foothill of Qinling-Daba Mountain
SSCP: single-stranded conformation polymorphism
SYR: the southern region of the Yangtze River
SZ: Sangzhi
TS: Tianshui
YS: Yongshun
