# Supplementary material for: Balancing selection and recombination as evolutionary forces caused population genetic variations in golden pheasant MHC class I genes
Source: BMC Evol Biol. 2016 Feb 18;16:42. doi: 10.1186/s12862-016-0609-0 (PMC4758006; doi:10.1186/s12862-016-0609-0)
Supplement: Additional file 1: Figure S1. — Network tree of exons 2 (A) and 3 (B) for golden pheasant MHC class I genes. (PDF 4 MB) [file 12862_2016_609_MOESM1_ESM.pdf]

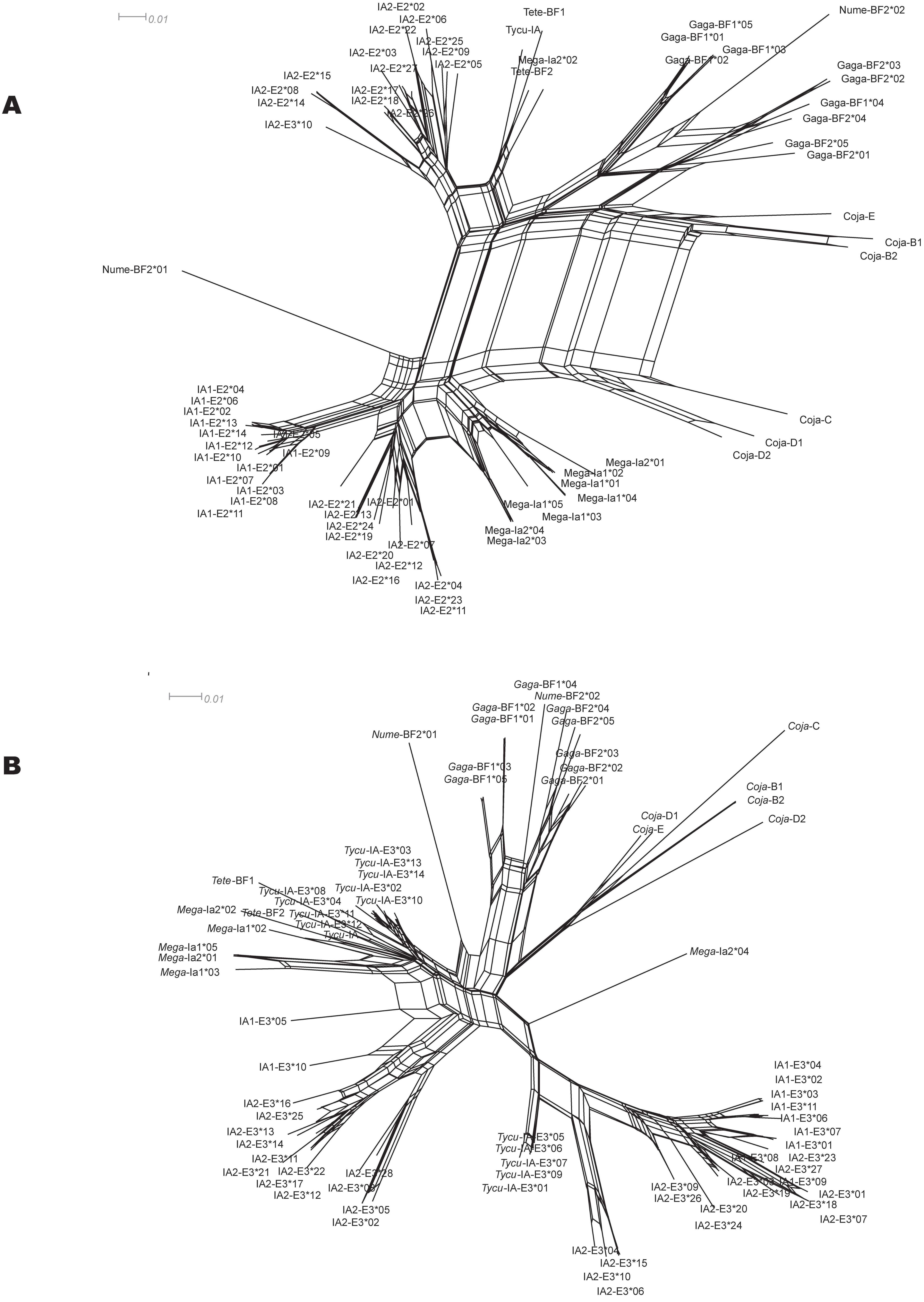

Figure S1 Network tree of exons 2 (A) and 3 (B) for golden pheasant MHC class I genes. Class I sequences from six other Galliformes birds listed in Figure 3 are also added to generate the tree.
