# Supplementary material for: Balancing selection and recombination as evolutionary forces caused population genetic variations in golden pheasant MHC class I genes
Source: BMC Evol Biol. 2016 Feb 18;16:42. doi: 10.1186/s12862-016-0609-0 (PMC4758006; doi:10.1186/s12862-016-0609-0)
Supplement: Additional file 2: Table S1. — Allele frequencies at MHC class I genes across golden pheasant populations. (PDF 611 kb) [file 12862_2016_609_MOESM2_ESM.pdf]

Table S1. Allele frequencies at MHC class I genes across golden pheasant populations

Locus	Allele	LX	TS	BJ	CQ	FN	JQ	LC	HN	QJ	GZ
**IA1**	*IA1*-E2*01	0.2	0.194	0.077	0.229	0.264	0.263	0.108	0.06		
	IA1-E2*02		0.097		0.071	0.014		0.04			
	IA1-E2*03	0.3	0.236	0.654	0.207	0.153	0.325	0.27	0.202	0.37	0.279
	IA1-E2*04		0.014		0.071	0.069		0.095	0.024		
	IA1-E2*05				0.071	0.055		0.041	0.06	0.087	0.029
	IA1-E2*06	0.3	0.292	0.154	0.257	0.306	0.312	0.176	0.369	0.13	0.236
	IA1-E2*07		0.014		0.029	0.069	0.05				0.029
	IA1-E2*08	0.05	0.069	0.077	0.022	0.028		0.189	0.202	0.195	0.25
	IA1-E2*09	0.15	0.042	0.038	0.036	0.042	0.012		0.023	0.196	0.133
	IA1-E2*10							0.081	0.06		0.044
	IA1-E2*11		0.014				0.038				
	IA1-E2*12		0.014		0.007						
	IA1-E2*13		0.014								
	IA1-E2*14									0.022	
	IA1-E3*01	0.333	0.208	0.115	0.321	0.389	0.288	0.135	0.036	0.065	0.03
	IA1-E3*02	0.222	0.389	0.115	0.336	0.361	0.275	0.23	0.452	0.087	0.25
	IA1-E3*03		0.069		0.129	0.139	0.05	0.108	0.048	0.283	0.147
	IA1-E3*04				0.014			0.014			
	IA1-E3*05	0.445	0.306	0.769	0.2	0.083	0.362	0.378	0.381	0.565	0.529
	IA1-E3*06					0.028		0.095	0.071		0.044
	IA1-E3*07		0.014					0.014			
	IA1-E3*08						0.025		0.012		
	IA1-E3*09							0.013			
	IA1-E3*10							0.013			
	IA1-E3*11		0.014								
**IA2**	*IA2*-E2*01		0.014		0.043	0.029			0.012	0.024	0.029
	IA2-E2*02	0.15	0.071		0.057	0.014	0.025		0.024		
	IA2-E2*03	0.1	0.243	0.167	0.25	0.243	0.113	0.189	0.22	0.357	0.456
	IA2-E2*04	0.4	0.243	0.083	0.221	0.286	0.45	0.135	0.171	0.119	0.103
	IA2-E2*05		0.029	0.125	0.179	0.157	0.162	0.027	0.122		0.015
	IA2-E2*06				0.021						
	IA2-E2*07					0.057			0.012		
	IA2-E2*08		0.029		0.014		0.013		0.012	0.048	0.029
	IA2-E2*09		0.029		0.007						

IA2-E2*10		0.029		0.007			0.014			
IA2-E2*11		0.029		0.007						
IA2-E2*12	0.05	0.057		0.014	0.029	0.088	0.135	0.098	0.119	0.088
IA2-E2*13		0.014		0.014		0.038	0.027		0.048	0.029
IA2-E2*14		0.014		0.014						
IA2-E2*15				0.007	0.014	0.013				
IA2-E2*16	0.05	0.029	0.375	0.021	0.014					
IA2-E2*17				0.014			0.014	0.024		
IA2-E2*18	0.2	0.1	0.083	0.057	0.086					
IA2-E2*19	0.05	0.057		0.007	0.057	0.025	0.122		0.167	0.177
IA2-E2*20				0.007		0.013	0.08		0.024	
IA2-E2*21				0.014			0.122	0.024	0.047	
IA2-E2*22		0.014		0.014	0.014		0.04	0.159		0.029
IA2-E2*23							0.015		0.047	
IA2-E2*24				0.007		0.037	0.08	0.11		0.015
IA2-E2*25						0.013				0.015
IA2-E2*26			0.167					0.012		0.015
IA2-E2*27		0.029								
IA2-E3*01	0.2	0.278	0.318	0.268	0.236	0.18	0.176	0.25	0.357	0.47
IA2-E3*02		0.056		0.203	0.041	0.18	0.027	0.092		0.015
IA2-E3*03	0.3	0.208	0.046	0.166	0.25	0.435	0.095	0.105	0.024	0.015
IA2-E3*04		0.015		0.015						
IA2-E3*05	0.1	0.028		0.015	0.014			0.026		0.06
IA2-E3*06	0.15	0.028		0.044	0.056	0.038		0.013		
IA2-E3*07		0.014		0.015		0.013		0.013		
IA2-E3*08	0.05	0.056	0.09	0.015	0.028	0.064	0.175	0.105	0.143	0.075
IA2-E3*09	0.05	0.097	0.046	0.043	0.055	0.012		0.013	0.024	0.015
IA2-E3*10				0.036			0.014			
IA2-E3*11		0.015		0.022			0.04	0.013	0.024	0.015
IA2-E3*12	0.05	0.042	0.046	0.007	0.014	0.025	0.04	0.04	0.048	
IA2-E3*13				0.015	0.014		0.122	0.013		
IA2-E3*14				0.007				0.013		
IA2-E3*15				0.015	0.014		0.014			
IA2-E3*16		0.028	0.364	0.007	0.014		0.014	0.013		
IA2-E3*17				0.014	0.028					
IA2-E3*18	0.05	0.056	0.045	0.035	0.055	0.025	0.175	0.119	0.261	0.197
IA2-E3*19	0.05	0.014	0.045	0.021	0.014		0.068		0.095	0.045
IA2-E3*20		0.015		0.015	0.028			0.013		
IA2-E3*21				0.015	0.083	0.014		0.04		0.015

IA2-E3*22	0.028			0.041	0.014	0.027	0.079		0.03
IA2-E3*23									0.015
IA2-E3*24							0.026		0.03
IA2-E3*25	0.014			0.015					
IA2-E3*26						0.013		0.024	
IA2-E3*27	0.014								
IA2-E3*28			0.007				0.014		
