# Supplementary material for: Balancing selection and recombination as evolutionary forces caused population genetic variations in golden pheasant MHC class I genes
Source: BMC Evol Biol. 2016 Feb 18;16:42. doi: 10.1186/s12862-016-0609-0 (PMC4758006; doi:10.1186/s12862-016-0609-0)
Supplement: Additional file 3: Table S2. — Summary of MHC genetic diversity in golden pheasant populations. (PDF 365 kb) [file 12862_2016_609_MOESM3_ESM.pdf]

Table S2. Summary of MHC genetic diversity in golden pheasant populations

Locus			LX[a] (10)	TS[a] (35)	BJ[a] (12)	CQ[a] (70)	FN[a] (36)	JQ[a] (40)	LC[b] (37)	HN[b] (42)	QJ[b] (23)	GZ[b] (34)
IA1	-E2	**R/A**	4.9/5	6.5/11	4.5/5	6.7/10	6.5/9	4.4/6	6.7/8	5.9/8	5.2/6	5.4/7
		H_O	0.7	0.838	0.538	0.771	0.833	0.65	0.703	0.659	0.696	0.706
		H_E	0.795	0.822	0.557	0.819	0.802	0.733	0.815	0.777	0.762	0.783
	-E3	**R/A**	3.0/3	4.3/6	3.0/3	4.2/5	4.2/5	4.0/5	5.7/9	4.1/6	3.7/4	4.0/5
		H_O	0.444	0.541	0.308	0.614	0.777	0.625	0.622	0.571	0.521	0.5
		H_E	0.679	0.715	0.397	0.727	0.701	0.719	0.764	0.649	0.602	0.633
IA2	-E2	**R/A**	6.7/7	8.8/16	5.9/6	8.2/22	7.2/12	6.7/13	8.6/13	7.7/13	7.5/10	6.6/12
		H_O	0.9	0.689	0.769	0.771	0.571	0.6	0.811	0.731*	0.651	0.735
		H_E	0.8	0.845	0.834	0.852	0.824	0.755	0.897	0.855	0.742	0.753
	-E3	**R/A**	8.5/9	9.0/18	7.1/8	8.5/22	9.1/18	5.9/11	8.5/14	9.5/19	6.3/9	6.7/13
		H_O	0.8	0.686	0.769	0.657*	0.724	0.7	0.837*	0.737	0.809	0.719
		H_E	0.837	0.851	0.827	0.861	0.853	0.749	0.887	0.88	0.84	0.75

Notes: Population abbreviations are described in Figure 1. Because sample numbers were limited, the Ningshan population was included within its neighboring population, Foping; similarly, the Qingchuan population was included within Jiange. For simplicity, both combinations are abbreviated as FN and JQ, respectively. Numbers in parentheses refer to population sample size. A: number of alleles; R: allelic richness; H_O: the observed heterozygosity; H_E: the expected heterozygosity; [a]: populations north of the Yangtze River; [b]: populations south of the Yangtze River; *: a significant departure from Hardy-Weinberg equilibrium after indicative Bonferroni adjusted nominal level (5%) for one population was 0.005.
