# Supplementary material for: Balancing selection and recombination as evolutionary forces caused population genetic variations in golden pheasant MHC class I genes
Source: BMC Evol Biol. 2016 Feb 18;16:42. doi: 10.1186/s12862-016-0609-0 (PMC4758006; doi:10.1186/s12862-016-0609-0)
Supplement: Additional file 4: Figure S2. — Results of Bayesian clustering analysis performed in STRUCTURE. (PDF 166 kb) [file 12862_2016_609_MOESM4_ESM.pdf]

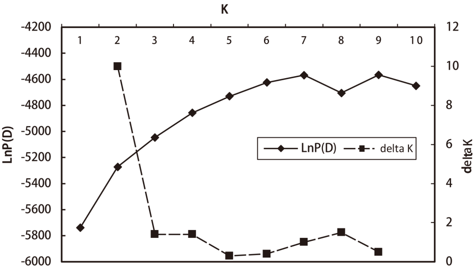

Figure S2 Results of Bayesian clustering analysis performed in STRUCTURE. For each K tested, the Ln P (D) equals the mean of the estimated log of the probability of the data across 10 different runs, while the ΔK was determined by the second order rate of change of these log likelihoods.
