# Supplementary material for: Balancing selection and recombination as evolutionary forces caused population genetic variations in golden pheasant MHC class I genes
Source: BMC Evol Biol. 2016 Feb 18;16:42. doi: 10.1186/s12862-016-0609-0 (PMC4758006; doi:10.1186/s12862-016-0609-0)
Supplement: Additional file 5: Table S3. — Pairwise F ST values among populations in MHC class I genes. (PDF 261 kb) [file 12862_2016_609_MOESM5_ESM.pdf]

Table S3. Pairwise *F_{ST}* values among populations in MHC class I genes

		Northern Yangtze Region						Southern Yangtze Region			
		**LX**	**TS**	**BJ**	**CQ**	**FN**	**JQ**	**LC**	**HN**	**QJ**	**GZ**
**Northern Yangtze Region**	**LX**	—	0.001	***0.103***	0.023	0.024	0.002	0.039	0.087	0.049	0.086
	**TS**	NS	—	***0.091***	0.007	0.015	0.024	0.022	0.063	0.019	0.046
	**BJ**	*	***	—	***0.126***	***0.161***	***0.139***	***0.08***	***0.103***	***0.105***	***0.103***
	**CQ**	NS	NS	***	—	0.004	0.027	0.043	0.093	0.041	0.08
	**FN**	NS	NS	***	NS	—	0.03	0.058	0.113	0.052	0.104
	**JQ**	NS	NS	***	NS	NS	—	0.063	0.129	0.062	0.122
**Southern Yangtze Region**	**LC**	*	*	***	***	***	***	—	0.031	0.026	0.036
	**HN**	***	***	***	***	***	***	NS	—	0.059	0.009
	**QJ**	*	*	***	***	***	***	NS	NS	—	0.04
	**GZ**	**	***	***	***	***	***	NS	NS	NS	—

Notes: Pairwise F_{ST} values and statistical significance levels and are shown above and below the diagonal, respectively. *: $P < 0.01$; **: $P < 0.001$; ***: $P < 0.0001$; NS: not significant ($P > 0.01$). Bold italics represent estimates of F_{ST} between BJ and any other population, which are statistically significant.
