# Supplementary material for: Balancing selection and recombination as evolutionary forces caused population genetic variations in golden pheasant MHC class I genes
Source: BMC Evol Biol. 2016 Feb 18;16:42. doi: 10.1186/s12862-016-0609-0 (PMC4758006; doi:10.1186/s12862-016-0609-0)
Supplement: Additional file 6: Figure S3. — Locus-specific primers amplifying exons 2 and 3 of golden pheasant MHC class I genes. (PDF 2 MB) [file 12862_2016_609_MOESM6_ESM.pdf]

A

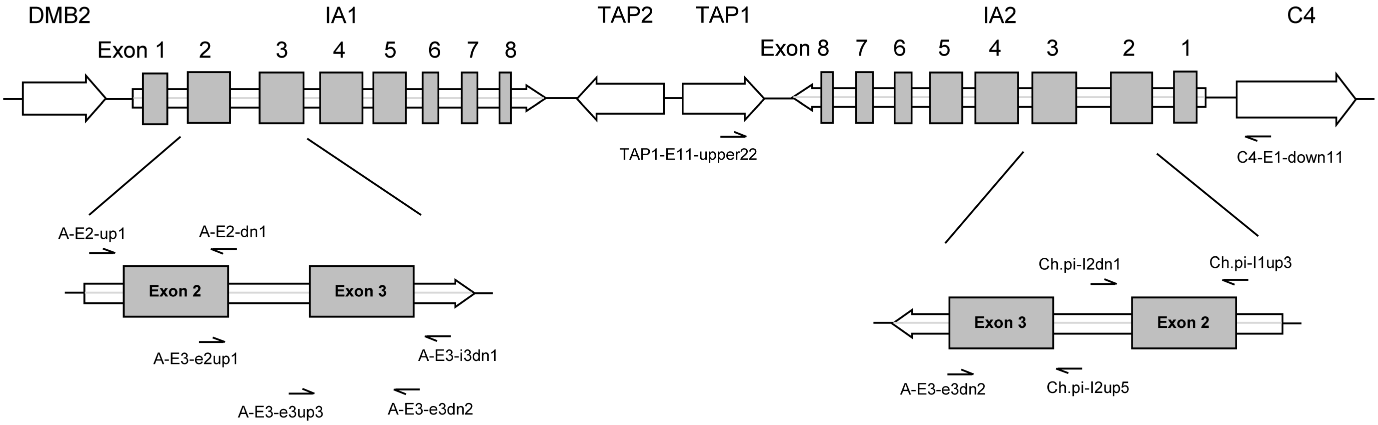

B

	Primer name	Primer sequence (5'-3')	Size (bp)	Ta (°C)
IA1-E2	A-E2-up1	TAACCCCAGCCCGGGGCTGT	346	55
	A-E2-dn1	ACCCCTGCTCTGGTTATAC		
IA1-E3	A-E3-e2up1[a]	GAACGGTATAACCAGAGCAGG	578	62
	A-E3-i3dn1[a]	GGCTGAGCTCCCCACGCT		
IA1-E3	A-E3-e3up3[b]	GTCTCACACATTGCAG	272	55
	A-E3-e3dn2[b]	TCCTGCCCAGCTCTGCCTTC		
IA2-E2/E3	TAP1-E11-upper22[a]	CACGAGGTGCTGCGCCCCGGCAG	4200	68
	C4-E1-down11[a]	CTCAGCCCATGCAGTCACCGTC		
IA2-E2	Ch.pi-I1up3[b]	CAGAGCCACACTCCCTGCGGTA	309	60
	Ch.pi-I2dn1[b]	CGGCACTGCGCCATGGAG		
IA2-E3	Ch.pi-I2up5[b]	CACCCCCTGCCCGCTBTGT	297	60

Figure S3 Locus-specific primers amplifying exons 2 and 3 of golden pheasant MHC class I genes. (A) Schematic representation of primer positions. Genes within the MHC class I genomic region are marked with white arrows denoting their transcriptional orientation, and gene names are listed above the diagram. For IA1 and IA2, exons and their flanking introns are illustrated by dark grey boxes and light grey lines, respectively. Primers are symbolized by small one-headed arrows indicating their positions and directions. On the left side of the lower part of the drawing, primers amplifying the target fragments of IA1 (Exon 2: A-E2-up1 and A-E2-dn1; Exon 3: the first round of nested PCR, A-E3-e2up1 and A-E3-i3dn1, and the second round, A-E3-e3up3 and A-E3-e3dn2) are given. On the right side of the lower part of the drawing, the first-round PCR primers which amplify the whole IA2 gene (TAP1-E11-upper22 and C4-E1-down11) are first given; Further down, the second-round primers targeting Exon 2 (Ch.pi-I1up3 and Ch.pi-I2dn1) and Exon 3 (Ch.pi-I2up5 and A-E3-e3dn2) are successively given. (B) List of primer sequences. In this diagram, the target product size and the annealing temperature of each amplification reaction are also provided. [a] and [b] correspond to primer pairs of the first and second round in the nested PCR, respectively.
